# Individual and interaction effects of health determinants on health-related quality of life in Korean adults aged 50–81 years: A causal Bayesian network analysis

**DOI:** 10.1371/journal.pone.0342187

**Published:** 2026-02-09

**Authors:** Chae Young Lee, Man-Suk Oh

**Affiliations:** Department of Statistics, Ewha Womans University, Seoul, Korea; Japanese Red Cross Medical Center, JAPAN

## Abstract

Health-related quality of life (HRQoL) reflects physical and mental well-being and is increasingly important in aging populations, yet traditional approaches often fail to capture the complex causal pathways among its determinants. We analyzed 2,566 adults aged 50–81 years from the Korean Genome and Epidemiology Study using the Short Form-12 (Physical Component Summary [PCS] and Mental Component Summary [MCS]). A causal Bayesian network was learned using the PC algorithm of Spirtes and Glymour with hierarchical constraints to ensure causal interpretability. We then estimated the causal effects of each variable on tail-defined outcomes—*poor* (bottom quartile) and *good* (top quartile) PCS and MCS—and quantified pairwise interaction effects. The network revealed how upstream factors propagate through direct and indirect pathways to shape HRQoL. Notably, PCS and MCS shared common upstream causes but showed no direct causal connection. Quantifying these causal pathways through relative risk (RR) estimates revealed the magnitude of individual factor effects. For poor PCS, severe insomnia (RR = 1.98), high stress (RR = 1.45), low physical activity (RR = 1.39), and multimorbidity (RR = 1.36) were the principal risk factors. For poor MCS, high stress (RR = 3.28) and severe insomnia (RR = 2.72) dominated. Notably, low BMI increased poor MCS risk (RR = 1.20), consistent with frailty pathways. The patterns for good outcomes largely mirrored these findings, with favorable levels showing protective effects. Interaction analyses revealed substantial synergistic effects: severe insomnia with high stress increased poor MCS probability by 6.44 percentage points (pp) beyond additivity, while high stress with physical inactivity added 4.77 pp. For good MCS, low insomnia with low stress (+4.72 pp) and low BMI with exercise (+4.21 pp) showed synergy, whereas stress with inactivity exhibited antagonism (–4.00 pp). These results support integrated interventions that combine sleep improvement, stress reduction, physical activity promotion, and multimorbidity management to improve HRQoL in aging populations.

## Introduction

Quality of life (QoL) is a multidimensional construct that reflects an individual’s perception of overall well-being across physical, psychological, and social domains [1]. Within this framework, Health-Related Quality of Life (HRQoL) has gained prominence as a critical measure in both clinical and public health contexts, as it captures the combined impact of physical and mental health on daily functioning and life satisfaction [2–4]. HRQoL is especially relevant in aging societies, where the management of chronic conditions and maintenance of mental health are increasingly important [5–7].

The Short Form-12 Health Survey (SF-12) is one of the most widely used instruments for measuring HRQoL [8]. It yields two composite indices: the Physical Component Summary (PCS) and the Mental Component Summary (MCS). These scores provide standardized assessments that facilitate population-level comparisons and guide healthcare strategies. Understanding how demographic, socioeconomic, lifestyle, medical, and psychological factors interact to shape PCS and MCS is therefore essential for developing effective interventions [9,10].

Traditional analytical approaches—including multiple regression models, structural equation modeling (SEM), and longitudinal mixed models—have consistently identified demographic factors (age, sex, income), health behaviors (exercise, smoking), chronic disease burden, and psychological distress as correlates of lower PCS and MCS scores [11–13]. Studies using the SF-12 or similar instruments in Korean cohorts have followed these conventional methods, documenting similar associations in older Korean adults [7,14–17]. However, these approaches are fundamentally limited in three key respects: they cannot distinguish direct from indirect causal pathways, they do not systematically model and test interaction terms to quantify synergistic or antagonistic effects among risk factors, and they provide associational rather than intervention-relevant effect estimates.

Bayesian networks (BNs) offer a complementary probabilistic graphical modeling framework that represents multivariable dependence structures as a directed acyclic graph (DAG), enabling both direct and indirect pathways to be visualized and quantified. A key advantage of BNs is their flexibility in incorporating prior knowledge—for example, through constraints that exclude implausible causal directions—thereby enhancing the plausibility and interpretability of the learned structures [18]. When combined with causal structure learning algorithms such as the PC algorithm [19], BNs can support causal interpretation under identification assumptions. These features make BNs particularly well suited for HRQoL research, where multifactorial influences and indirect pathways are central, and where distinguishing association from intervention-relevant effects is crucial.

Recent applications have demonstrated the utility of BNs in HRQoL research across diverse contexts. Several studies have successfully applied BNs to map between different HRQoL instruments [20,21], forecast long-term post-surgical outcomes [22], and explore associational structures among symptoms and quality of life in clinical populations [23]. For instance, Le and Doctor [21] used BNs to probabilistically convert SF-12 responses into EQ-5D utility indices in a US sample, while Cao et al. [22] employed Gaussian BNs to predict 5-year HRQoL scores following bariatric surgery in Scandinavian patients. Methodological advances have further demonstrated the feasibility of causal structure learning in HRQoL contexts: Ganopoulou et al. [24] systematically compared five constraint-based algorithms for learning causal structures from discrete ordinal HRQoL data, demonstrating that algorithm performance depends critically on sample size and structural complexity, while Ganopoulou et al. [25] proposed leveraging differences in learned causal structures between patient groups for classification purposes. However, existing BN applications to HRQoL have not estimated causal effects of modifiable determinants or quantified their interaction effects in general populations.

In this study, we applied the PC algorithm with bootstrap aggregation [26] to data from the Ansan cohort of the Korean Genome and Epidemiology Study (KoGES) [27]. To ensure causal interpretability, we implemented a hierarchical blacklist encoding a life-course ordering (demographic → socioeconomic → behaviors/psychological → medical → HRQoL). Our analysis focused on identifying modifiable determinants—such as physical activity, regular exercise, insomnia, stress, and multimorbidity—that influence PCS and MCS scores. We estimated causal effects via do-interventional probabilities for each variable and investigated interaction effects among risk factors. This approach complements and extends prior HRQoL studies by providing an explicit causal graph, do-interventional (policy-relevant) effect estimates, and pairwise interaction assessments within a single coherent framework— thereby moving beyond traditional associational analyses to deliver evidence for integrated intervention strategies that target the most influential and synergistic determinants of HRQoL in older Korean adults.

## Materials and methods

### Study population and data sources

The study protocol was reviewed by the Ewha Womans University Institutional Review Board (IRB No. 2024-0079) and determined to be exempt on March 27, 2024; the IRB also waived informed consent because this secondary analysis used de-identified data and posed minimal risk. The authors had no access to personally identifiable information. Data were provided by the Clinical & Omics Data Archive (CODA) of the Korea Disease Control and Prevention Agency (KDCA) under approval (CODA_S2400014-01), with a permitted use period through 2026-02-28. Data were accessed for research purposes on 14/06/2024.

This study used data from the Ansan cohort of the Korean Genome and Epidemiology Study (KoGES), conducted by the National Institute of Health under the KDCA. All original cohort participants provided written informed consent, and de-identified genetic and health information were supplied to the authors via CODA.

The study population comprises residents of Ansan, an industrialized city located approximately 32 km southwest of Seoul (population ∼670,000 as of October 2024). Centered around the Banwol Industrial Complex, Ansan has been considered representative of Korean urban populations, as documented in prior KoGES-based studies [28,29]. The Ansan cohort was initiated in 2001, with biennial follow-ups thereafter.

For the present analysis, we used data from the 7th follow-up survey (2013–2014), which includes the most recent SF-12 responses from n=2,689 participants. After excluding individuals with missing information, the final analytic sample comprised n=2,566 participants aged 50–81 years.

### SF-12 health survey

The SF-12 Health Survey is a widely used standard instrument for assessing HRQoL [30–32]. The KoGES dataset includes responses to the 12-item SF-12 version 2 questionnaire, which can typically be completed in under two minutes, making it highly suitable for large-scale epidemiological studies.

The 12 items of the SF-12 consist of 10 questions on a 5-point Likert scale and 2 questions on a 3-point Likert scale, covering eight domains: physical functioning (PF1, PF2), role limitations due to physical health (RP1, RP2), role limitations due to emotional problems (RE1, RE2), bodily pain (BP), general health (GH), vitality (VT), social functioning (SF), and mental health (MH1, MH2). These domains are summarized into two composite scores: PCS and MCS, which serve as the target variables in this study.

PCS and MCS were computed using norm-based scoring methods, which apply factor scoring coefficients derived from the 1998 U.S. general population [33]. This approach is widely adopted because it facilitates cross-country comparisons and interpretation using standardized benchmarks.

The scoring process involves two steps. First, weighted aggregate scores are calculated by applying factor scoring coefficients to the standardized item scores of the eight SF-12 domains. The weighted scoring formulas are shown below:

PCS=0.42402×GH+0.35119×PF1+0.48907×PF2+0.15714×RP1+0.17835×RP2+0.28343×BP−0.09731×VT−0.04695×SF−0.00753×RE1+0.01553×RE2−0.20963×MH1−0.10531×MH2,MCS=−0.22999×GH−0.10337×PF1−0.09731×PF2−0.01483×RP1−0.11454×RP2−0.07311×BP+0.23534×VT+0.26876×SF+0.21060×RE1+0.18602×RE2+0.48581×MH1+0.43407×MH2. (1)

Second, these aggregate scores are transformed into T-scores with a mean of 50 and a standard deviation of 10, based on the 1998 U.S. general population norms. This standardization ensures comparability across populations and allows scores to be interpreted relative to a nationally representative reference group.

### Variables

Target variables were PCS and MCS, as they can be measures of physical and mental component of HRQoL, respectively.

Candidate variables influencing HRQoL were selected based on previous studies [34–36]. General characteristics included sex (SEX), age (AGE), marital status (MARRY), employment status (JOB), and monthly income (INCOME), which are known to shape subjective well-being [37,38]. Body mass index (BMI) was also included as an essential factor due to its well-documented association with HRQoL [39,40].

Chronic conditions were represented by the number of co-existing chronic non-communicable diseases (nCNCD) among arthritis, hypertension, diabetes, and allergies—conditions frequently identified as key determinants of HRQoL [14,15,41,42].

Lifestyle factors included regular exercise (EXER), alcohol consumption (DRINK), smoking status (SMOKE), total physical activity volume per day (ACT), insomnia severity (INSOMNIA), and perceived stress (PSS), all of which have been shown to influence HRQoL [16,43–45].

ACT represents total daily physical activity volume (metabolic hours per day) calculated using the metabolic equivalent of task [46]. It was computed as the time-weighted sum of intensity-specific metabolic values based on reported time in each activity category [47]:


ACT=1.0×(sedentary time)+1.5×(very light activity time)+2.5×(light activity time)+4.5×(moderate activity time)+7.0×(vigorous activity time).

INSOMNIA was measured using the Insomnia Severity Index (ISI) [48], while perceived stress was assessed using the Perceived Stress Scale [49]. For both scales, lower scores indicate more favorable outcomes. Age and monthly income, originally continuous, were categorized to enhance model interpretability in BN learning: age into three levels (54–55,55–69,70–81 years) and monthly household income into three levels (<1, 1−4, ≥4 million KRW), labeled as Low, Mid, and High.

### Learning a causal Bayesian network

A BN is a type of machine learning model that combines Bayesian theory and graph theory to model dependency relationships among variables. It is a probabilistic graphical model that calculates conditional probabilities between variables using Bayes’ theorem and visualizes their interactions through a DAG [50].

A DAG consists of nodes, which represent variables, and arcs (edges), which depict the conditional independence relationships between variables. The arcs are directed from parent nodes to child nodes, indicating that each variable is conditionally probabilistically dependent on its parent variables in the DAG.

A BN satisfies the Markov condition, which allows the joint probability distribution of the nodes to be decomposed into a product of conditional probability distributions, as follows:


$$P(X_{1},\ldots ,X_{p})=\prod_{i=1}^{p}P(X_{i}|\Pi_{i}).$$

Here, $𝐗=\{X_{1},\ldots ,X_{p}\}$ represents the set of *p* random variables, and $\Pi_{i}$ denotes the parent nodes of *X*_*i*_ [51–53]. This decomposition automates inference and learning in BNs, significantly enhancing computational efficiency [54].

Employing the ability of BNs to incorporate domain-specific information in structure learning, we imposed a blacklist to exclude implausible paths and thereby enhance the causal interpretability of the network. A blacklisted arc represents a relationship that is prohibited from appearing in the learned DAG [54]. To enforce logical temporal and etiological ordering, five hierarchical layers were defined (Table 1), and arcs directed from higher-numbered layers to lower-numbered layers were prohibited. This ordering encodes a life-course perspective: fixed demographic attributes, followed by socio-economic conditions, modifiable health behaviors, medical burden, and finally health-related quality of life (HRQoL).

**Table 1:** Variable layers used to impose blacklist in a Bayesian network.

Layer	Group	Variables
Layer 1	Demographic	SEX, AGE
Layer 2	Socio-economic	MARRY, JOB, INCOME
Layer 3	Health/Psychological	BMI, EXER, DRINK, SMOKE, ACT, INSOMNIA, PSS
Layer 4	Medical condition	nCNCD
Layer 5	HRQoL	PCS, MCS

Layer 1 included sex (SEX) and age (AGE), which are exogenous determinants that cannot be caused by any study variable. To avoid encoding their empirical correlation as a spurious directed arc, connections between SEX and AGE were further restricted. Layer 2 contained socio-economic variables (MARRY, JOB, INCOME), which shape opportunities and constraints for health but are not credibly caused by downstream behaviors in a cross-sectional framework. Layer 3 consisted of health behaviors and psychosocial variables (BMI, EXER, DRINK, SMOKE, ACT, INSOMNIA, PSS), which are proximate risk factors shaped by demographics and socioeconomic status and were thus positioned after Layer 2. Layer 4 included medical conditions (nCNCD), reflecting accumulated morbidity that develops primarily downstream of long-term behaviors and stress exposures. Finally, Layer 5 comprised the outcomes of interest (PCS, MCS), representing end states of physical and mental health functioning.

This hierarchical blacklist also serves several methodological purposes. It reduces the likelihood of implausible feedback loops (e.g., PCS→SMOKE), constrains the search space to enhance model stability, and ensures that causal directions align with substantive epidemiologic knowledge. Overall, the layered blacklist regularizes structure learning while preserving the causal interpretability of the resulting BN.

Given this blacklist, we applied the PC algorithm [19] to learn the remaining network structure from the data. The PC algorithm is a widely used constraint-based method for causal discovery in BN construction. Like all causal inference methods applied to observational data, it relies on explicit causal identification assumptions [55,56]: (1) *causal sufficiency*—all common causes of the observed variables are measured and included; (2) *consistency*—the observed outcome under a given exposure equals the potential outcome under that exposure; and (3) *positivity*—all exposure levels occur with positive probability in the population. Under these assumptions, the PC algorithm identifies the underlying causal structure up to a *Markov equivalence class* [55,56]—a set of DAGs that encode identical conditional independence relationships and therefore cannot be statistically distinguished. For example, the causal chains A → B → C and A ← B ← C both imply that A and C are conditionally independent given B, placing them in the same equivalence class.

Among these assumptions, causal sufficiency is particularly challenging to ensure in observational health research, where unmeasured factors such as genetic predispositions, early-life experiences, or lifestyle behaviors may confound multiple relationships. Our hierarchical blacklist addresses this challenge by incorporating established domain knowledge—temporal sequences and etiological pathways from life-course epidemiology—to constrain the learned structure. This hybrid approach combines the strengths of domain expertise (ruling out implausible causal directions) with statistical learning (discovering empirically supported relationships within those constraints), thereby reducing the risk of spurious arcs due to unmeasured confounding while allowing the data to inform the final network structure.

In this study, we employed a variation of the original PC algorithm known as the PC-stable algorithm, which was developed to improve the reliability and consistency of arc discovery during structure learning [57]. The network was estimated using the pc.stable function implemented in the *bnlearn* package in R [58].

A single dataset may offer unstable BN model structure. To get around this, we employed bootstrap aggregation (bagging) to bolster confidence [26]. From the dataset, we created 15,000 new datasets (called bootstrap samples) of the size equal to 80% of the original dataset size by randomly sampling with replacement. The BN model was then trained on each of these samples.

Based on the 15,000 BNs, we calculated the arc strength and direction for each arc. The arc strength is the relative frequency of the arc’s presence across the networks, regardless of direction. The arc direction is the relative frequency of the arc’s specific direction across the networks. The arc strength represents the relative magnitude of dependency between two variables, while the arc direction distinguishes parent and child nodes [59,60]. Both arc strength and direction are expressed as values between 0 and 1, with values closer to 1 indicating stronger strength and directionality.

Using model averaging, a consensus network was constructed by including only arcs with arc strengths exceeding a specified threshold to eliminate weak or spurious connections and retain only robust relationships. Higher thresholds correspond to sparser networks.

## Results

### Characteristics of the study participants

Table 2 summarizes the study sample characteristics. Pairwise association tests were conducted between each variable and the target variables (PCS and MCS). For continuous variables, we assessed monotonic associations using Spearman’s rank correlation; for categorical variables, group differences were evaluated with the Kruskal–Wallis test [61–63]. The corresponding p-values are reported in the last two columns of Table 2.

**Table 2:** Summary of study participants. Frequencies (%) are shown for categorical variables and Mean ± SD for continuous variables. p-values represent results from Kruskal–Wallis tests (categorical) and Spearman correlations (continuous) with PCS and MCS.

Variable	Description	Category	Mean ± SD	p-value (PCS)	p-value (MCS)
**Target Variable**					
PCS	Physical Component Summary		50.00 ± 10.00		<0.001
MCS	Mental Component Summary		50.00 ± 10.00	<0.001	
**Demographic factor**					
SEX	Sex	Male	1269 (49.5%)	<0.001	0.004
		Female	1297 (50.5%)		
AGE	Age	50~54	667 (26.0%)	<0.001	0.009
		55~69	1626 (63.4%)		
		70~81	273 (10.6%)		
**Socioeconomic factor**					
MARRY	Marital status	Yes (currently married)	2309 (90.0%)	0.002	0.177
		No (else)	257 (10.0%)		
JOB	Employment status	Yes (economic activity)	1547 (60.3%)	<0.001	0.059
		No (non-economic)	1019 (39.7%)		
INCOME	Average monthly income	Low (< 1)	229 (8.9%)	<0.001	0.107
	(million Korean won)	Mid (1≤ <4)	1381 (53.8%)		
		High (≥4)	956 (37.3%)		
**Health behavior**					
EXER	Regular exercise	No	883 (34.4%)	<0.001	0.001
		Yes	1683 (65.6%)		
DRINK	Alcohol consumption	No (Non-drinker)	1217 (47.4%)	<0.001	0.143
		Yes (Former/current drinker)	1349 (52.6%)		
SMOKE	Lifetime consumption	No (Non-smoker)	1587 (61.8%)	<0.001	0.947
	of ≥400 cigarettes	Yes (Current/former smoker)	979 (38.2%)		
BMI	Body mass index		24.52 ± 2.91	0.019	<0.001
ACT	Daily physical activity volume		42.71 ± 6.48	<0.001	0.484
INSOMNIA	Insomnia score		6.74 ± 5.07	<0.001	<0.001
PSS	Stress score		15.83 ± 5.08	<0.001	<0.001
**Medical condition**					
nCNCD	Number of chronic diseases		0.22 ± 0.53	<0.001	0.967

The results indicated that PCS was significantly associated with all variables at the 5% significance level, whereas MCS showed significant associations with SEX, AGE, EXER, BMI, INSOMNIA, and PSS. Additionally, PCS and MCS were correlated with each other.

### Causal Bayesian network

In examining the results obtained by varying the arc strength threshold used for BN model averaging, we found that the number of included arcs differed substantially between thresholds of 0.1 and 0.2, whereas increasing the threshold from 0.2 to 0.3, 0.4, and 0.5 resulted in the number of arcs decreasing by two at each step. To achieve a more precise identification of associations while accounting for the modest sample size (n = 2,566), we selected a threshold of 0.3 for constructing the final BN model in this study.

**Fig 1:**
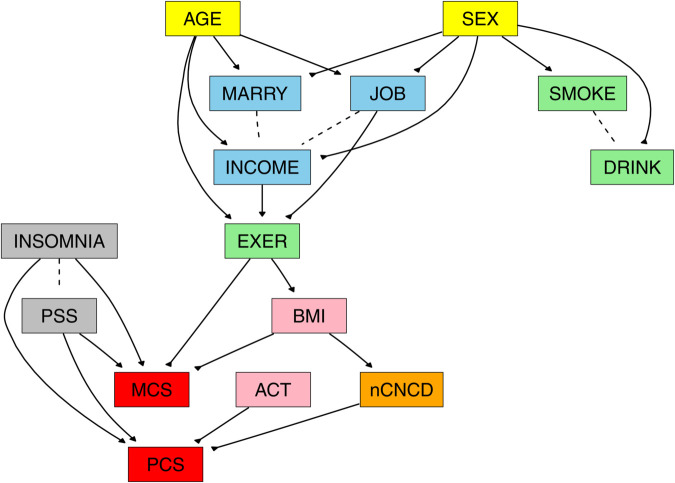
Causal Bayesian network for health-related quality of life in Korean adults aged 50–81 years. The directed acyclic graph represents causal relationships among determinants of physical (PCS) and mental (MCS) health-related quality of life. Nodes represent variables, and directed arcs indicate causal pathways from parent to child nodes. Dashed lines denote variable pairs with directional uncertainty (arc directions 0.51–0.57). Variables are color-coded by domain: demographic (yellow: SEX, AGE), socioeconomic (blue: MARRY, JOB, INCOME), lifestyle behaviors (green: EXER, DRINK, SMOKE), anthropometric/physical activity (pink: BMI, ACT), psychological (gray: INSOMNIA, PSS), medical conditions (orange: nCNCD), and HRQoL outcomes (red: PCS, MCS). **Abbreviations:** ACT, daily physical activity volume; AGE, age; BMI, body mass index; DRINK, alcohol consumption; EXER, regular exercise; INCOME, monthly household income; INSOMNIA, insomnia severity; JOB, employment status; MARRY, marital status; MCS, Mental Component Summary; nCNCD, number of chronic non-communicable diseases; PCS, Physical Component Summary; PSS, perceived stress; SEX, sex; SMOKE, lifetime consumption of ≥400 cigarettes.

The final DAG is presented in Fig 1. In the figure, demographic factors are shown in yellow; socioeconomic factors in blue; lifestyle factors (EXER, DRINK, SMOKE) in green; anthropometric and physical activity factors (BMI, ACT) in pink; psychological factors (INSOMNIA, PSS) in gray; medical condition (nCNCD) in orange; and HRQoL outcomes (PCS, MCS) in red.

All arcs in Fig 1 showed high directional stability (arc directions >0.9) except for four pairs—SMOKE–DRINK, MARRY–INCOME, JOB–INCOME, and INSOMNIA–PSS—which exhibited low directional stability (arc directions 0.51–0.57). Arc directions close to 0.5 indicate that the data provide insufficient information to reliably determine the direction of effect. Such instability may arise from unmeasured common causes, simultaneous determination, or potentially reciprocal (bidirectional) relationships that cannot be represented within an acyclic DAG framework. Accordingly, we treat these links as non-directional associations and depict them as dashed lines in Fig 1.

### Individual effects

Based on the causal structure in Fig 1, we quantified the interventional effects of each variable on both HRQoL components—PCS and MCS—using tail-specific outcomes as causal targets. We defined *poor* PCS/MCS as values below the 25th percentile and *good* PCS/MCS as values above the 75th percentile, and then estimated how intervening on each variable shifts the probabilities of these lower- and upper-tail events.

We demonstrate the calculation of interventional probability of poor PCS as an illustrative example. For each variable *X*, we estimated the interventional probability of poor PCS as


P(poor PCS∣do(X=x)),

where *x* denotes a specified level of variable *X*. For a continuous variable, *X* was categorized into three levels (Low, Mid, High) using the 25th and 75th percentiles as cut points.

The do-operator do(X=x), introduced by Pearl [56] and further developed by Hernán and Robins [55], estimates interventional probabilities by artificially fixing *X* at level *x* while disconnecting *X* from its parent nodes in the network, thereby removing potential confounding influences. Unlike conditional probabilities, P(Y∣X=x), which reflect mere associations, interventional probabilities, P(Y∣do(X=x)), isolate the causal effect of *X* on *Y* and answer the policy-relevant question of “what happens if we intervene” rather than “what happens when we observe” [64].

The critical distinction between conditional and interventional probabilities can be illustrated with PCS and MCS. Although Fig 1 contains no connection between PCS and MCS, they are *correlated in the observed data* (Table 2), resulting in different conditional probabilities: P(poor MCS∣poor PCS)≠P(poor MCS∣non-poor PCS). This discrepancy arises because conditional probabilities reflect associations induced by shared causes rather than a direct causal effect of PCS on MCS [56,64]. By contrast, the interventional query P(poor MCS∣do(PCS=p)) asks what would happen if PCS were externally set to a level *p* while the rest of the network evolved according to the learned causal structure [56]. Under this framework, the estimates of P(poor MCS∣do(PCS=poor)) and P(poor MCS∣do(PCS=non-poor)) were equal, indicating that PCS itself exerts *no causal effect* on MCS despite their observed association.

Among the variables involved in the four pairs with uncertain directions, INSOMNIA and PSS exhibited substantially different interventional effects depending on the assumed direction between them, whereas the other variables (SMOKE, DRINK, MARRY, JOB, INCOME) showed negligible interventional effects on both PCS and MCS regardless of their assumed directions.

Because INSOMNIA and PSS are the primary determinants of HRQoL in our analysis, we conducted a sensitivity analysis to incorporate directional uncertainty in the INSOMNIA–PSS link. Specifically, we fitted two networks: BN_insomnia (with INSOMNIA → PSS enforced) and BN_pss (with PSS → INSOMNIA enforced). The two networks were structurally identical except for this single arc direction. Individual-effect estimates for poor/good PCS and poor/good MCS were computed from each network (S1–S4 Figs). To account for directional uncertainty, we calculated final estimates as weighted averages using the arc directions (0.56 for INSOMNIA → PSS and 0.44 for PSS → INSOMNIA) as weights.

Importantly, the estimated effects of all other variables remained identical across the two networks. This invariance occurs because the pair INSOMNIA–PSS have no parent nodes in the learned network (Fig 1); therefore, reversing the INSOMNIA–PSS arc direction does not alter the parent sets—and hence the causal pathways—of any other variables in the DAG.

**Fig 2:**
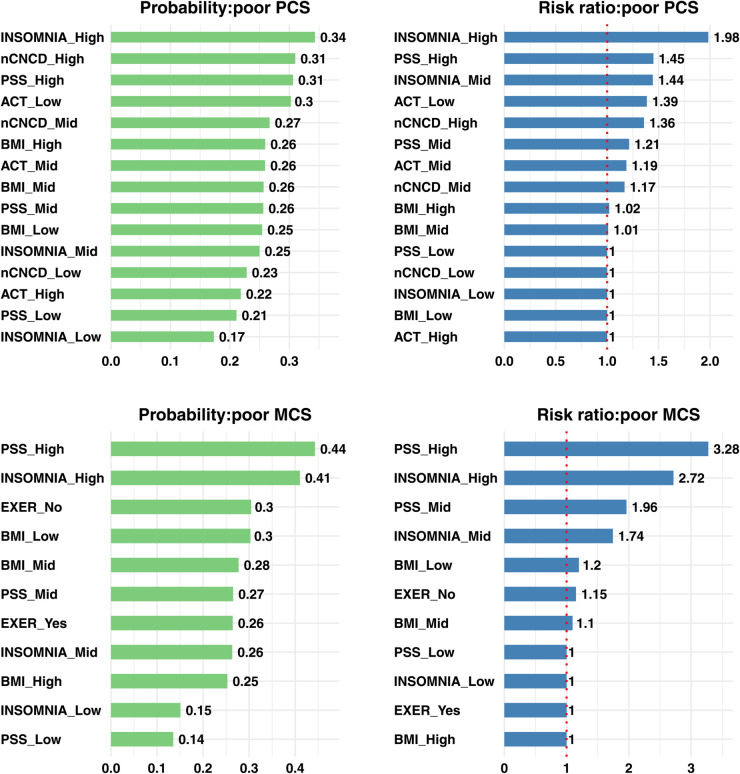
Estimated interventional probabilities and risk ratios of poor health-related quality of life. Top row: Physical Component Summary (PCS); bottom row: Mental Component Summary (MCS). Left panels show interventional probabilities of scores below the 25th percentile (poor HRQoL). Right panels show risk ratios relative to the reference category, with the vertical dotted line marking RR = 1. For INSOMNIA and PSS, estimates are weighted averages from BN_insomnia_ (INSOMNIA→PSS, weight 0.56) and BN_pss_ (PSS→INSOMNIA, weight 0.44). **Abbreviations:** as in Fig 1.

**Fig 3:**
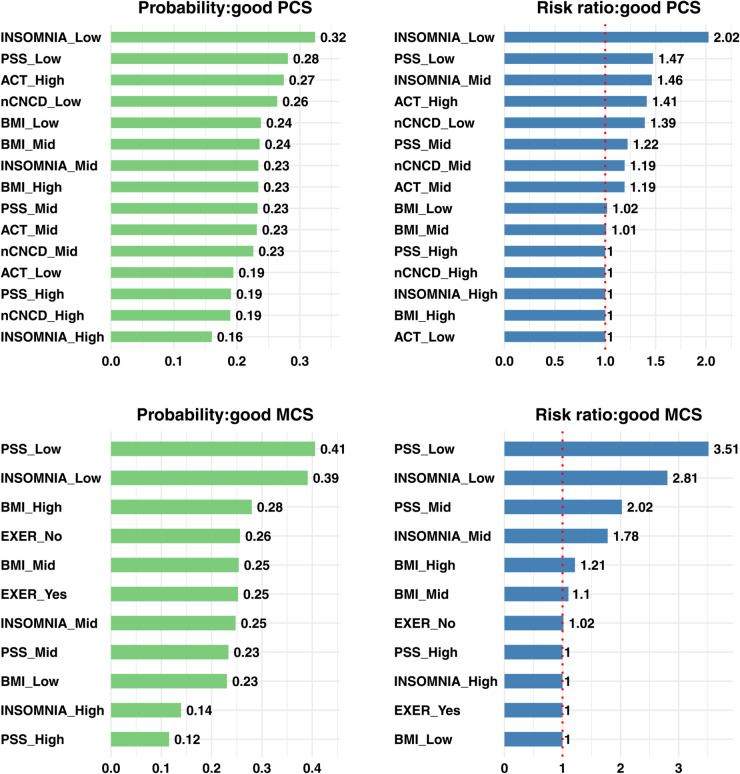
Estimated interventional probabilities and risk ratios of good health-related quality of life. Top row: Physical Component Summary (PCS); bottom row: Mental Component Summary (MCS). Left panels show interventional probabilities of scores above the 75th percentile (good HRQoL). Right panels show risk ratios relative to the reference category, with the vertical dotted line marking RR = 1. For INSOMNIA and PSS, estimates are weighted averages from BN_insomnia_ (INSOMNIA→PSS, weight 0.56) and BN_pss_ (PSS→INSOMNIA, weight 0.44). **Abbreviations:** as in Fig 1.

Using these weighted estimates, we quantified the interventional effects of all variables directly or indirectly influencing HRQoL. Figs 2 and 3 display the estimated interventional probabilities and risk ratios (RRs) for poor and good HRQoL, respectively. The RR represents the interventional probability relative to the reference level. To enhance interpretability, we excluded variables for which the probabilities were nearly identical across levels.

Examining both tails of the HRQoL distribution provides a more comprehensive understanding of causal dynamics. Lower-tail analyses (poor PCS/MCS, below 25th percentile) identify risk factors that contribute to HRQoL deterioration, whereas upper-tail analyses (good PCS/MCS, above 75th percentile) reveal protective or enhancing factors that promote high functioning. This dual characterization is essential for intervention design: the determinants that prevent decline into poor HRQoL may differ in magnitude or even direction from those that actively foster attainment of excellent HRQoL, and effective population-level strategies should address both prevention and promotion.

### Interaction effects

Analysis of interaction effects among risk factors clarifies whether joint exposures produce outcomes that depart from the additivity of their individual effects. Using the do-interventional framework, we quantified pairwise interactions for PCS and MCS on both additive and relative scales. This approach enabled identification of high-risk exposure combinations and provided an evidence base for targeted, multi-component interventions that may more effectively improve HRQoL than single-factor strategies.

For simplicity, all continuous variables were dichotomized at the 75th percentile to yield binary levels (0,1), with level 1 indicating the higher-risk category for adverse outcomes. Let *p*_*ab*_ denote the probability of an outcome when Var1=a and Var2=b (a,b∈{0,1}), where the outcome may be *poor* PCS/MCS or *good* PCS/MCS. Coding was chosen so that, for each variable, level 1 corresponds to a higher probability of the adverse outcome than level 0. The sole exception was physical activity (ACT), for which lower values imply poorer HRQoL; accordingly, ACT was dichotomized at the 25th percentile so that level 1 indicates low activity and level 0 high activity. The same notation was used for the “good” outcomes (good PCS/MCS), with variable codings unchanged; consequently, level 1 entailed a lower probability of the good outcome.

The additive interaction effect is defined as


$$\text{Interaction}=p_{11}-p_{10}-p_{01}+p_{00},$$

where a positive value indicates *synergy* (the combined effect exceeds the sum of individual contributions), while a negative value reflects *antagonism* (partial cancellation of effects).

The total effect is expressed as


$$\text{Total effect}=p_{11}-p_{00},$$

and the relative interaction,


Relative interaction=Interaction/Total effect,

captures the magnitude of interaction relative to the overall combined effect [65,66].

Tables 3 and 4 summarize the additive and relative interaction effects on poor and good HRQoL, respectively. To ensure interpretability, we reported only variable pairs for which both the absolute additive interaction and the absolute total effect exceeded 1 percentage point (pp) (i.e., >0.01 in probability units).

**Table 3:** Additive interaction, total effect, and relative interaction for selected risk-factor pairs on poor PCS and poor MCS. For each pair, interaction and total effect are weighted averages (and ranges) across BN_insomnia (INSOMNIA→PSS) and BN_pss (PSS→INSOMNIA) with weights 0.56 and 0.44, respectively. Relative interaction is the ratio of additive interaction to total effect.

Target	Var1	Var2	Interaction (min, max)	Total effect (min, max)	Relative interaction
Poor PCS	INSOMNIA	ACT	0.0120 (0.0113, 0.0126)	0.1832 (0.1751, 0.1896)	0.0656
	INSOMNIA	nCNCD	0.0117 (0.0110, 0.0122)	0.1465 (0.1402, 0.1514)	0.0797
	INSOMNIA	PSS	0.0111	0.1719	0.0646
Poor MCS	INSOMNIA	PSS	0.0644	0.2938	0.2193
	PSS	EXER	0.0477 (0.0465, 0.0493)	0.2339 (0.2218, 0.2494)	0.2040
	BMI	PSS	0.0141 (0.0128, 0.0158)	0.2318 (0.2200, 0.2468)	0.0609
	BMI	INSOMNIA	0.0128 (0.0122, 0.0136)	0.1885 (0.1672, 0.2052)	0.0680

**Table 4:** Additive interaction, total effect, and relative interaction for selected risk-factor pairs on good PCS and good MCS. For each pair, interaction and total effect are weighted averages (and ranges) across BN_insomnia (INSOMNIA→PSS) and BN_pss (PSS→INSOMNIA) with weights 0.56 and 0.44, respectively. Relative interaction is the ratio of additive interaction to total effect.

Target	Var1	Var2	Interaction (min, max)	Total effect (min, max)	Relative interaction
Good PCS	INSOMNIA	ACT	0.0150 (0.0141, 0.0157)	0.1493 (0.1436, 0.1537)	0.1005
	INSOMNIA	PSS	0.0139	0.1413	0.0984
	INSOMNIA	nCNCD	0.0104 (0.0098, 0.0108)	0.1610 (0.1531, 0.1672)	0.0643
Good MCS	INSOMNIA	PSS	0.0472	0.3763	0.1254
	BMI	EXER	0.0421	0.0156 (0.0155, 0.0156)	2.7056
	PSS	EXER	–0.0400 (–0.0409, –0.0385)	0.2134 (0.1981, 0.2333)	–0.1859
	BMI	PSS	0.0135 (0.0125, 0.0147)	0.2487 (0.2336, 0.2679)	0.0542

Interaction estimates’ sensitivity to the direction betwen INSOMNIA and PSS depends on which variables are intervened. For the INSOMNIA–PSS pair, both variables are simultaneously intervened, which removes all incoming arcs to both nodes—including their mutual arc—thereby rendering the direction between them irrelevant. Consequently, interaction estimates for INSOMNIA–PSS are identical across BN_insomnia and BN_pss. Similarly, pairs not involving INSOMNIA or PSS are unaffected by the INSOMNIA–PSS arc direction because reversing this arc does not alter the parent sets of other variables in the network.

In contrast, for pairs involving only one of INSOMNIA or PSS (e.g., INSOMNIA–ACT, PSS–EXER), the assumed direction matters. Under INSOMNIA → PSS, intervening on INSOMNIA can affect PSS through the directed arc, whereas under PSS → INSOMNIA, it cannot. These differing causal pathways yield direction-dependent interaction estimates. For such direction-sensitive pairs, we computed weighted averages of the additive interactions and total effects from BN_insomnia and BN_pss, using the arc directions 0.56 and 0.44, respectively, for as weights. Relative interactions were then calculated as the ratio of the weighted average interaction to the weighted average total effect. To convey sensitivity to the assumed arc direction, we report both the weighted average and the range (minimum and maximum across the two directions) for additive interactions and total effects.

## Discussion

Using BN analysis with causal structure learning, we identified key modifiable determinants of physical and mental quality of life in older Korean adults from the KoGES Ansan cohort. For physical health (PCS), severe insomnia, high stress, and low physical activity were the principal modifiable risk factors. For mental health (MCS), high perceived stress and severe insomnia showed substantially stronger effects, with their joint presence producing synergistic effects. Notably, while PCS and MCS shared common upstream determinants, no direct causal link connected them. Additionally, low BMI was adversely associated with MCS, consistent with frailty pathways in this mid-to-older adult cohort.

Our findings align with prior research on HRQoL determinants in Korean populations while providing novel causal insights. Studies using regression or SEM approaches have identified demographic factors, health behaviors, chronic disease burden, and psychological distress as correlates of lower PCS and MCS [7,14–17]. By applying causal structure learning, we move beyond association to identify intervention-relevant pathways—distinguishing direct effects from those mediated through intermediate variables—with immediate implications for designing integrated interventions.

### Causal network structure

We found that the learned network conformed to the prespecified hierarchy (demographic → socioeconomic → health/medical → HRQoL) and clarified how upstream factors propagate to physical (PCS) and mental (MCS) quality of life. Age and sex sat at the top of the graph, shaping marital status, employment, and income. Socioeconomic position then influenced regular exercise (EXER), indicating that exercise was, in part, conditioned by socioeconomic resources. Sex also governed smoking and drinking. These behaviors remained largely peripheral once upstream determinants were accounted for, suggesting that their direct effects on HRQoL were limited or operated mainly through intermediates in this cohort.

Among the lifestyle factors, EXER played a central role. There was an EXER → BMI → multimorbidity (nCNCD) → PCS pathway, consistent with a sequence in which regular exercise improves body composition and reduces disease burden, thereby enhancing physical functioning [67,68]. There was no dominant direct arc from BMI to PCS; rather, BMI’s influence appeared to operate primarily through nCNCD, aligning with literature that emphasizes functional capacity and disease burden over weight per se in older adults [69–72]. Total physical activity volume per day (ACT) had a direct arc to PCS but was otherwise largely isolated from other nodes, indicating a second, independent conduit to physical functioning (ACT → PCS) that is distinct from the EXER → BMI → nCNCD → PCS route.

The psychological cluster showed that insomnia (INSOMNIA) and perceived stress (PSS) each sent arcs to both MCS and PCS, indicating cross-domain impacts of sleep and stress on mental and physical HRQoL. The learned network did not include a direct arc between MCS and PCS, implying that observed associations between the two domains are largely explained by shared upstream determinants rather than a direct causal link.

### Individual effects

The interventional analysis based on Fig 2 identified a small set of modifiable factors that substantially influence the deterioration of HRQoL.

For poor PCS, severe insomnia was the strongest determinant, with a probability of poor PCS of about 0.34 compared to 0.17 under low insomnia, corresponding to RR of 1.98. High perceived stress (PSS_High) yielded a probability of 0.31 (RR 1.45), high multimorbidity (nCNCD_High) 0.31 (RR 1.36), low physical activity (ACT_Low) 0.30 (RR 1.39). In contrast, BMI categories clustered near 0.25–0.26 with RRs close to 1.0, suggesting negligible influence. Overall, PCS impairment in this cohort was primarily driven by insomnia, stress, multimorbidity, and inactivity with BMI and regular exercise contributing little, a pattern consistent with these dominant factors accounting for most of the variation under the do(·)-interventional analysis.

For poor MCS, psychological determinants were dominant. High stress increased the probability of poor MCS to 0.44 (vs. 0.14 under low stress), corresponding to RR=3.28. Severe insomnia increased the probability to 0.41 (vs. 0.15 under low insomnia), corresponding to RR=2.72. Moderate stress (probability 0.27; RR 1.96) and mid-level insomnia (probability 0.26; RR 1.74) showed clear dose–response patterns. Lifestyle and anthropometric factors had smaller but nontrivial effects: lack of regular exercise (EXER_No) raised the probability to 0.30 (RR 1.15), and low BMI likewise to 0.30 (RR 1.20). Notably, *low*—rather than high—BMI emerged as a risk factor for poorer MCS. This pattern aligns with the cohort’s mid-to-older age structure, in which underweight often reflects frailty, illness-related weight loss, and stress/sleep dysregulation—pathways more directly linked to mental well-being [73–75]. In contrast, excess weight appeared neutral or weakly protective after adjustment [76–78].

Three cross-domain patterns are evident. First, sleep disturbance and stress substantially worsen both PCS and MCS, with particularly pronounced effects on MCS. Second, physical activity volume (ACT) and regular exercise (EXER) influence health differently: low activity volume primarily harms PCS, whereas lack of regular exercise affects MCS. Third, multimorbidity has stronger effects on PCS, while low BMI is more relevant for MCS. Importantly, demographic and socioeconomic variables exert minimal direct influence once downstream mediators are accounted for.

Fig 3 presents the complementary analysis for good outcomes (top-quartile PCS and MCS). As expected, the directions largely mirror the low-tail results. For PCS, better sleep (INSOMNIA_Low) most strongly increased the interventional probability and RR of achieving good physical health. High physical activity (ACT_High), low multimorbidity, and low stress raised the chance of top-quartile PCS. For MCS, low stress and better sleep most strongly increased the interventional probability and RR of achieving good mental health. High BMI had a moderate effect on good mental health. However, regular exercise had negligible effect on good MCS, which contrasts with the adverse effect of no regular exercise (RR 1.15) on poor MCS.

Across both PCS and MCS, the inclusion of good-outcome analyses reinforces that the same modifiable factors govern the entire HRQoL spectrum—that is, they shift risk *across the full distribution*, from reducing the probability of falling into the lower tail (poor outcomes, e.g., <25th percentile) to increasing the chance of attaining the upper tail (good outcomes, e.g., >75th percentile). In practical terms, improving sleep and lowering stress, together with maintaining higher activity levels, appears to move individuals along this continuum rather than merely pushing them across a single threshold, yielding both prevention of decline and promotion of high functioning. Domain-specific leverage also differs: activity volume and multimorbidity exert more influence on PCS, whereas stress and insomnia dominate MCS.

### Interaction effects

Table 3 summarizes the additive and relative interaction effects for poor HRQoL outcomes. Because ratio-based measures can exaggerate magnitude when total effects are small, we prioritized additive interactions for interpretation and reported relative measures for completeness.

For PCS, additive interactions were generally modest, with a few reproducible signals: INSOMNIA×ACT (+1.20 pp), INSOMNIA×PSS (+1.17 pp), and INSOMNIA×nCNCD (+1.11 pp) slightly increased the probability of poor PCS beyond additivity.

For MCS, interactions were more pronounced. The strongest signal was INSOMNIA×PSS (+6.44 pp), underscoring the synergistic impact of sleep disturbance and perceived stress on mental health. PSS×EXER (+4.77 pp) also showed a substantial positive interaction, indicating that high stress combined with no regular exercise elevated risk beyond the sum of their individual effects. Additional moderate interactions included BMI×PSS (+1.41 pp), BMI×INSOMNIA (+1.28 pp).

Given that higher BMI was associated with better MCS in our cohort, BMI was dichotomized at the 25th percentile for interaction analysis on MCS, with low BMI (below the 25th percentile) coded as the risk level (level = 1). This approach ensured that the risky category comprised 25% of the population, matching the dichotomization strategy applied to other variables and facilitating direct comparability across all pairwise interactions.

The interaction pattern suggests that mental health risks in this cohort are disproportionately concentrated among individuals with co-occurring sleep disturbance and perceived stress. The large positive additive interaction for INSOMNIA×PSS indicates that addressing either factor in isolation may leave a substantial portion of excess risk unmitigated; their joint presence produces more poor-MCS cases than expected from summing individual effects. Likewise, PSS × EXER implies that inactivity amplifies the mental-health burden of stress. Moderate interactions with BMI (BMI × PSS, BMI × INSOMNIA) further point to a cluster in which sleep, stress, and weight status jointly shape mental well-being. In contrast, PCS interactions are small, suggesting that physical health risks are driven more by independent contributions than by strong synergies between pairs.

Table 4 presents the analogous results for *good* HRQoL outcomes. Overall, good-tail interactions closely matched the poor-tail patterns. For good PCS, the additive interactions for INSOMNIA×PSS and INSOMNIA×nCNCD were of similar magnitude to those observed for poor PCS.

For good MCS, the INSOMNIA×PSS interaction was attenuated relative to poor MCS, whereas BMI×INSOMNIA and BMI×PSS were broadly comparable across outcome definitions.

Although interaction signs need not mirror each other because good and poor MCS target different tails (75th vs. 25th percentiles), it was notable that PSS×EXER switched sign—from +4.77 pp for poor MCS to –4.00 pp for good MCS— indicating an antagonistic interaction for the good outcome. Recall that EXER=1 denotes *no* regular exercise and EXER=0 denotes regular exercise. Under high stress (PSS=1), regular exercise modestly improved the probability of good MCS ($p_{10}\;-\;p_{11}=0.010$), whereas under low stress (PSS=0), regular exercise counterintuitively reduced this probability ($p_{00}\;-\;p_{01}=-0.030$). Thus, exercise yielded a small improvement under high stress but a comparatively larger reduction under low stress, suggesting that the benefit of regular exercise for achieving good MCS is stress-dependent. Because similar results were obtained even after changing the dichotomization cut point for PSS, the pattern is unlikely to be driven by distributional artifacts. Moreover, when we extended the analysis to three-way interaction effects—including variables such as INSOMNIA and BMI—the findings remained consistent, suggesting that the variables used in this study are not producing a confounding effect. We therefore hypothesize that unobserved characteristics—such as good sleep quality, positive attitudes, strong social networks, or psychological resilience—may be disproportionately present in the low-stress & no-exercise group and could account for the observed pattern. Further research is warranted to investigate these latent factors.

In summary, the large positive additive interactions for INSOMNIA × PSS and PSS × EXER on poor MCS indicate that the combined burden of these factors exceeds the sum of their individual effects, underscoring synergistic pathways in mental health deterioration.

### Practical implications

The causal network and interventional estimates suggest that insomnia (INSOMNIA) and perceived stress (PSS) are high-leverage, modifiable determinants with direct impacts on both PCS and MCS, and that adverse outcomes are amplified when these factors co-occur or coincide with inactivity. These findings support routine screening in primary care for adults over 50 using brief validated instruments (e.g., ISI for insomnia and PSS-4 [79] for stress), followed by a staged support pathway that links positive screens to scalable options (sleep-hygiene counseling, brief CBT-I elements, stress-management skills training) with referral when indicated. When insomnia and high stress co-occur (or high stress accompanies inactivity), the interaction patterns favor coordinated plans that address both drivers rather than isolated, single-target management.

At the population level, the results point to community programs that pair sleep, stress, and physical-activity components, reflecting the central roles of insomnia/stress and the combinations associated with poorer HRQoL—especially poorer MCS. Practical options include community sleep-health education, stress-reduction workshops, and accessible physical-activity opportunities (group classes, supervised sessions, and initiatives to reduce sedentary time).

Finally, the network’s socioeconomic-to-behavior channel (e.g., INCOME/JOB → EXER) implies that sustained improvements in HRQoL may require structural supports that make behavior change feasible—such as affordable access to facilities, safe and walkable environments, transportation, and opportunities for social participation—particularly for economically disadvantaged older adults. While these interventional estimates are hypothesis-generating and not substitutes for randomized trials, they provide a principled basis to prioritize targets and design interaction-informed, multi-level strategies for aging populations.

### Limitations

Several limitations should be acknowledged. First, we imposed a hierarchical pathways (demographic → socioeconomic → health behaviors → medical conditions → HRQoL) to constrain admissible directions; although grounded in expert knowledge and guidelines, any a priori restriction and prior specification of arc directions can introduce subjectivity.

Second, causal discovery with the PC algorithm relies on standard identification assumptions—consistency, positivity, and causal sufficiency (no unmeasured confounding). Applied to cross-sectional observational data, violations of causal sufficiency could misorient within-layer arcs and limit our ability to establish causal directions or rule out reverse causation for specific pathways.

Third, we considered only the number of chronic diseases, even though their effects on HRQoL may vary by type. When we re-estimated the network including four specific chronic diseases as binary variables, they were connected only to each other and not to other factors. This likely reflects their low prevalence (<5%) in our community-based sample and the modest overall sample size, which limited power to detect disease-specific pathways and links to upstream determinants. Importantly, we lacked direct measures of chronic pain and clinical depression— both major determinants of HRQoL in older adults. While insomnia and perceived stress served as psychological indicators, these do not substitute for depression assessment. In disease-enriched settings or populations with higher prevalence of musculoskeletal conditions, chronic pain, or depression, we would expect stronger connections from specific diseases to PCS/MCS and tighter coupling with psychological factors such as sleep and stress.

Fourth, generalizability should be interpreted cautiously. Our results are based on a single community-based cohort of Korean adults aged 50–81 years, and both the learned network structure and the magnitudes of estimated interventional effects may differ in populations with different age distributions, healthcare systems, cultural norms, and disease burdens.

Finally, estimation of individual and interaction causal effects in this study required the discretization of continuous variables, which may have resulted in information loss and sensitivity of results to the chosen thresholds.

Given these limitations, our findings should be interpreted as cohort-specific, hypothesis-generating evidence for intervention prioritization rather than definitive policy prescriptions. Future studies should apply this approach to independent cohorts—including non-Korean populations, clinically enriched samples with richer disease and mental health phenotyping, and longitudinal studies that better support temporal ordering—to validate the estimated interventional effects and assess the transportability of the inferred causal structure. Formal transportability analyses comparing structural features and key interventional contrasts across diverse populations would further strengthen the evidence base for causal approaches to HRQoL research.

## Conclusions

A causal BN of HRQoL in Korean adults clarified how upstream factors propagate to physical and mental functioning and enabled policy-relevant interventional contrasts. Perceived stress and insomnia emerged as the most influential—and synergistic—determinants of MCS, while insomnia, multimorbidity, and low physical activity were the principal drivers of PCS. Low BMI was adverse for MCS, consistent with frailty pathways in mid-to-older age. Interaction analyses highlighted particularly strong joint effects of insomnia with stress and of stress with inactivity on poor mental health; conversely, for good outcomes, the low-stress, no-exercise subgroup exhibited unexpectedly high rates of good MCS—likely reflecting unmeasured protective factors. Despite strong pairwise association, PCS and MCS did not exhibit a direct causal link in the BN, suggesting their covariation is largely explained by shared upstream determinants.

## Supporting information

S1 Fig Estimated interventional probabilities and risk ratios of poor health-related quality of life from BN_insomnia (INSOMNIA→PSS). (EPS)

S2 Fig Estimated interventional probabilities and risk ratios of good health-related quality of life from BN_insomnia (INSOMNIA→PSS). (EPS)

S3 Fig Estimated interventional probabilities and risk ratios of poor health-related quality of life from BN_pss (PSS→INSOMNIA). (EPS)

S4 Fig Estimated interventional probabilities and risk ratios of good health-related quality of life from BN_pss (PSS→INSOMNIA). (EPS)
